# Supplementary material for: Role of c-Myc activating the transcription of PD-L1 in immune escape during benzene-induced malignant transformation of human lymphoblasts
Source: PLoS One. 2025 Sep 12;20(9):e0332277. doi: 10.1371/journal.pone.0332277 (PMC12431550; doi:10.1371/journal.pone.0332277)
Supplement: Attachment — Submitted filename: Dear Editors of PlOS One.docx [file pone.0332277.s009.docx]

Dear Editors of *PlOS One*

Thank you very much for your detailed review and valuable suggestions. We have carefully considered each point raised in your comments and have revised the manuscript accordingly. Below, we provide a point-by-point response:

Editor's comment 1:"Please provide more detailed information regarding the care and use of animals in the "Methods" section for the survival study, which must comply with our editorial guidelines."

Response 1:"We have provided more detailed information regarding the care and use of animals in the "Methods" section of the study, and have made modifications based on the checklist. The specific locations in the text have been noted on the checklist."

Editor's comment 2:"Please provide the magazine with the details of the ethical review and the specific methods of euthanasia.."

Response 2:"The Ethics Statement will be submitted through the "Other" option and the specific methods of euthanasia are presented in lines 155-157 of the manuscript."

We greatly appreciate the time and effort you have devoted to reviewing our work. Your insiglas and recommendatious have been istuneutal in improving the clarily and quality of our mauuscript. Should you have any further suggestious or roquire additional revisions, please do ot Lesitate to let us know. We look forward to your feedback and Lope that te revised version meets with your approval.

Sincerely

Huanwen Tang
