# Supplementary material for: Role of c-Myc activating the transcription of PD-L1 in immune escape during benzene-induced malignant transformation of human lymphoblasts
Source: PLoS One. 2025 Sep 12;20(9):e0332277. doi: 10.1371/journal.pone.0332277 (PMC12431550; doi:10.1371/journal.pone.0332277)
Supplement: Attachment — Submitted filename: Dear Editors and Reviewers of PlOS One.docx [file pone.0332277.s010.docx]

Dear Editors and Reviewers of *PlOS One:*

Thank you very much for your detailed review and valuable suggestions. We have carefully considered each point raised in your comments and have revised the manuscript accordingly. Below, we provide a response:

Thank the editor for reminding us that there are still some issues with the article. The editor mentioned that the main body of the article should not contain any financial information. We have remove any funding-related text from the manuscript revise. We have submitted the funding information that is presented in the "Funding Statement" section. Previously in the "Data Availability" section, we chose "Yes - all data are fully available without restriction". However, all relevant data will be included in the main paper and its supplementary information file. We sincerely apologize for the mistakes we made when choosing the options before.

We appreciate the reviewer's valuable feedback regarding language issues. We have made every effort to address grammatical concerns throughout the manuscript. Additionally, the formatting and reference style have been updated to comply with the the International Committee of Medical Journal Editors (ICMJE), also referred to as the “Vancouver” style (line 360-440 in the revised manuscript). However, as the reviewer did not specify the exact locations of grammatical errors, our revisions may not have captured all instances. We would be pleased to receive additional guidance should concerns persist."

**Reviewer's comment 1:**

" the statistical analysis isn't elaborate enough as a thorough search was needed on the readers' part for specific statistics. Additionally, conclusions and discussion could be elaborated in more details."

**Response 1:**

"We appreciate the reviewer's valuable feedback regarding language issues. We have made every effort to incorporate the relevant conclusions and discuss the content of the relevant sections.

1. Regarding the previous statistical methods, they have been corrected specifically (lines 170-173 in the revised manuscript).
2. Moreover, We expanded the discussion and closely related it to the results for further examination. We have also made a certain amount of expansion to the conclusion (lines 342-346 in the revised manuscript) and incorporated relevant content regarding the transformation of EBV DNA in the study of genomic stability and genetic toxicity into the discussion section (lines 278-288 in the revised manuscript)."

**Reviewer's comment 2:"Please provide**

Lines 107-108. Provide company name, city, & country of Reverse transcriptase.
Line 114. Provide company name, city, & country of RIPA buffer.
Line 156. Error? Or change the bold “reach the anesthetic dose).
Figures: The resolution for all the figures needs to be fixed."

**Response 2:**

"We appreciate the reviewer's valuable feedback regarding language issues. We have made every effort to relevant content throughout the manuscript.

1.We have made every effort to incorporate relevant supplementary content and formatting throughout the manuscript.Reverse transcription(Seville, Wuhan, China) (lines 112 in the revised manuscript). RIPA buffer (CST, Danvers, Massachusetts, USA) (lines 118-119 in the revised manuscript). The font error in line 157 of the previous article has been corrected (line 160 in the revised manuscript).

2.The figures regarding image resolution has standardized all the resolutions to a unified format."

We greatly appreciate the effort you have devoted to reviewing our work. Your insights and recommendations have been instrumental in improving the clarity and quality of our manuscript. Should you have any further suggestions or require additional revisions, please do not hesitate to let us know. We look forward to your feedback and hope that the revised version meets with your approval.

Sincerely,

Huanwen Tang
